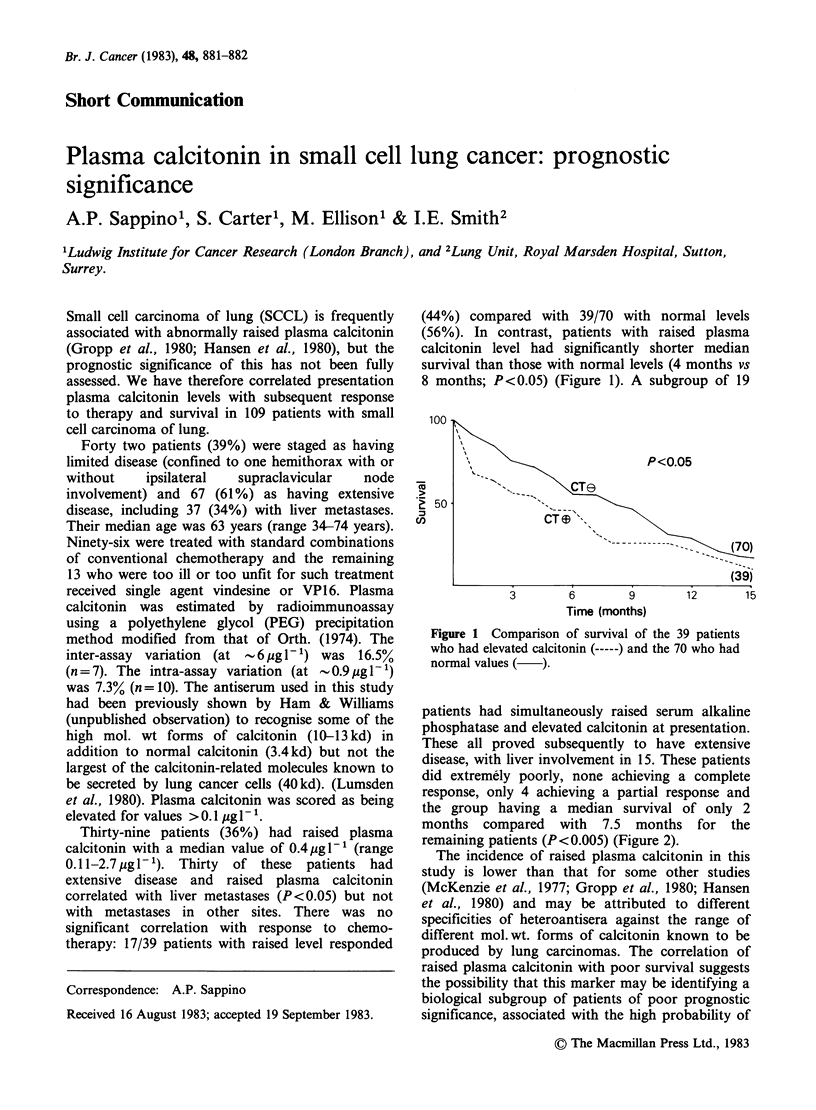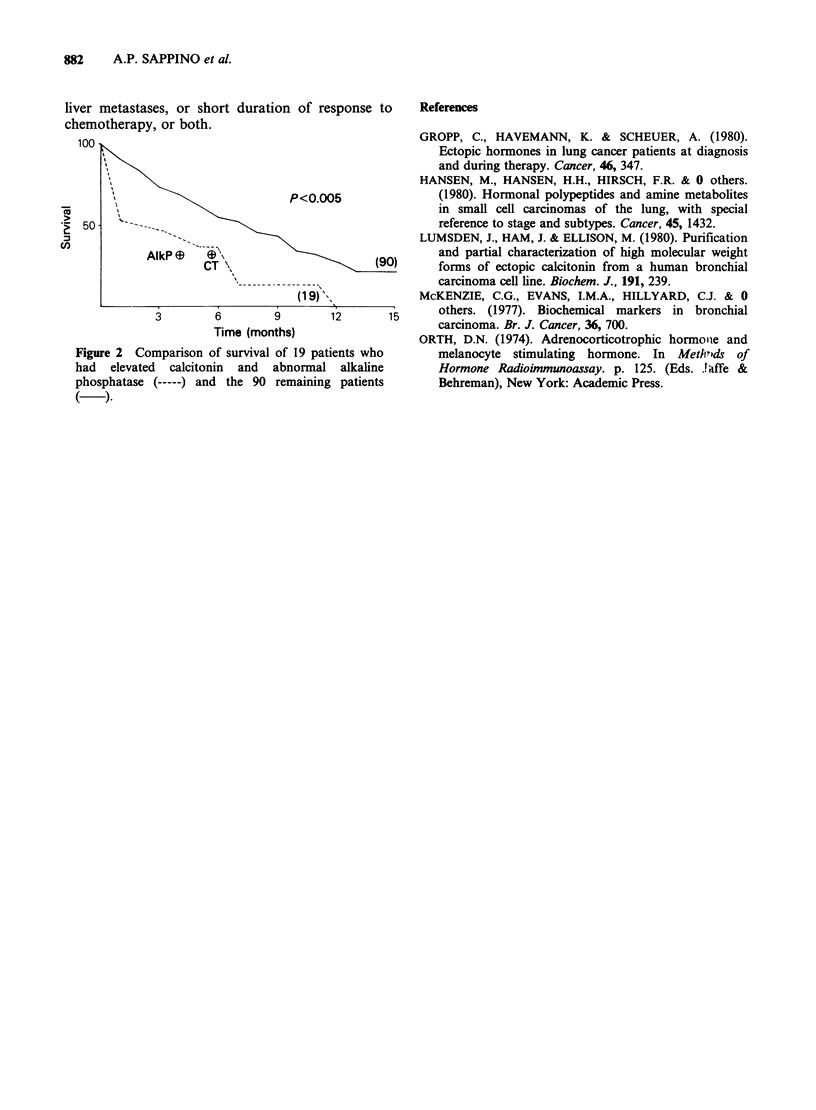# Plasma calcitonin in small cell lung cancer: prognostic significance.

**DOI:** 10.1038/bjc.1983.283

**Published:** 1983-12

**Authors:** A. P. Sappino, S. Carter, M. Ellison, I. E. Smith


*Br. J. Cancer* (1983), **48**, 881–882

### Short Communication

### Plasma calcitonin in small cell lung cancer: prognostic significance

A.P. Sappino[1], S. Carter[1], M. Ellison[1] & I.E. Smith[2]

[1]*Ludwig Institute for Cancer Research (London Branch), and* [2]*Lung Unit, Royal Marsden Hospital, Sutton, Surrey.*

Small cell carcinoma of lung (SCCL) is frequently associated with abnormally raised plasma calcitonin (Gropp *et al.*, 1980; Hansen *et al.*, 1980), but the prognostic significance of this has not been fully assessed. We have therefore correlated presentation plasma calcitonin levels with subsequent response to therapy and survival in 109 patients with small cell carcinoma of lung.

Forty two patients (39%) were staged as having limited disease (confined to one hemithorax with or without ipsilateral supraclavicular node involvement) and 67 (61%) as having extensive disease, including 37 (34%) with liver metastases. Their median age was 63 years (range 34–74 years). Ninety-six were treated with standard combinations of conventional chemotherapy and the remaining 13 who were too ill or too unfit for such treatment received single agent vindesine or VP16. Plasma calcitonin was estimated by radioimmunoassay using a polyethylene glycol (PEG) precipitation method modified from that of Orth. (1974). The inter-assay variation (at $\sim 6\,\mu g\,l^{-1}$) was 16.5% ($n=7$). The intra-assay variation (at $\sim 0.9\,\mu g\,l^{-1}$) was 7.3% ($n=10$). The antiserum used in this study had been previously shown by Ham & Williams (unpublished observation) to recognise some of the high mol. wt forms of calcitonin (10–13 kd) in addition to normal calcitonin (3.4 kd) but not the largest of the calcitonin-related molecules known to be secreted by lung cancer cells (40 kd). (Lumsden *et al.*, 1980). Plasma calcitonin was scored as being elevated for values $>0.1\,\mu g\,l^{-1}$.

Thirty-nine patients (36%) had raised plasma calcitonin with a median value of $0.4\,\mu g\,l^{-1}$ (range $0.11$–$2.7\,\mu g\,l^{-1}$). Thirty of these patients had extensive disease and raised plasma calcitonin correlated with liver metastases ($P<0.05$) but not with metastases in other sites. There was no significant correlation with response to chemotherapy: 17/39 patients with raised level responded (44%) compared with 39/70 with normal levels (56%). In contrast, patients with raised plasma calcitonin level had significantly shorter median survival than those with normal levels (4 months *vs* 8 months; $P<0.05$) (Figure 1). A subgroup of 19

**Figure 1** Comparison of survival of the 39 patients who had elevated calcitonin (-----) and the 70 who had normal values (——).

patients had simultaneously raised serum alkaline phosphatase and elevated calcitonin at presentation. These all proved subsequently to have extensive disease, with liver involvement in 15. These patients did extremely poorly, none achieving a complete response, only 4 achieving a partial response and the group having a median survival of only 2 months compared with 7.5 months for the remaining patients ($P<0.005$) (Figure 2).

The incidence of raised plasma calcitonin in this study is lower than that for some other studies (McKenzie *et al.*, 1977; Gropp *et al.*, 1980; Hansen *et al.*, 1980) and may be attributed to different specificities of heteroantisera against the range of different mol. wt. forms of calcitonin known to be produced by lung carcinomas. The correlation of raised plasma calcitonin with poor survival suggests the possibility that this marker may be identifying a biological subgroup of patients of poor prognostic significance, associated with the high probability of

Correspondence: A.P. Sappino

Received 16 August 1983; accepted 19 September 1983.

© The Macmillan Press Ltd., 1983

liver metastases, or short duration of response to chemotherapy, or both.

**Figure 2** Comparison of survival of 19 patients who had elevated calcitonin and abnormal alkaline phosphatase (-----) and the 90 remaining patients (——).

**References**

GROPP, C., HAVEMANN, K. & SCHEUER, A. (1980). Ectopic hormones in lung cancer patients at diagnosis and during therapy. *Cancer*, **46**, 347.

HANSEN, M., HANSEN, H.H., HIRSCH, F.R. & 0 others. (1980). Hormonal polypeptides and amine metabolites in small cell carcinomas of the lung, with special reference to stage and subtypes. *Cancer*, **45**, 1432.

LUMSDEN, J., HAM, J. & ELLISON, M. (1980). Purification and partial characterization of high molecular weight forms of ectopic calcitonin from a human bronchial carcinoma cell line. *Biochem. J.*, **191**, 239.

McKENZIE, C.G., EVANS, I.M.A., HILLYARD, C.J. & 0 others. (1977). Biochemical markers in bronchial carcinoma. *Br. J. Cancer*, **36**, 700.

ORTH, D.N. (1974). Adrenocorticotrophic hormone and melanocyte stimulating hormone. In *Methods of Hormone Radioimmunoassay*. p. 125. (Eds. Jaffe & Behreman), New York: Academic Press.